# Photodegradation of Rhodamine B over Biomass-Derived Activated Carbon Supported CdS Nanomaterials under Visible Irradiation

**DOI:** 10.3389/fchem.2017.00123

**Published:** 2017-12-20

**Authors:** Hai-Bo Huang, Yu Wang, Feng-Ying Cai, Wen-Bin Jiao, Ning Zhang, Cheng Liu, Hai-Lei Cao, Jian Lü

**Affiliations:** ^1^Fujian Provincial Key Laboratory of Soil Environmental Health and Regulation, College of Resources and Environment, Fujian Agriculture and Forestry University, Fuzhou, China; ^2^State Key Laboratory of Structural Chemistry, Fujian Institute of Research on the Structure of Matter, Chinese Academy of Sciences, Fuzhou, China

**Keywords:** biomass, activated carbon, photocatalysis, cadmium sulfide, composite materials

## Abstract

A family of new composite materials was successfully prepared through the deposition of as-synthesized CdS nanomaterials on lotus-seedpod-derived activated carbon (SAC). The SAC supports derived at different activation temperatures exhibited considerably large surface areas and various microstructures that were of great importance in enhancing photocatalytic performance of CdS@SAC composite materials toward the photodegradation of rhodamine B (RhB) under visible irradiation. The best-performing CdS@SAC-800 showed excellent photocatalytic activity with a rate constant of ca. 2.40 × 10^−2^ min^−1^, which was approximately 13 times higher than that of the CdS nanomaterials. Moreover, the estimated band gap energy of CdS@SAC-800 was significantly lowered down to 1.99 eV compared to that of the CdS precursor (2.22 eV), which suggested considerable strength of interface contact between the CdS and SAC support, as well as efficient light harvesting capacity of the composite material. Further photocatalytic study indicated that the SAC supports enhanced the separation of photogenerated electrons and holes in this system. Improved photocatalytic activity of the composite materials was largely due to the increased generation of catalytically active species such as *h*^+^, OH•, ${\text{O}}_{2}{•}^{-}$ etc. This work provided a facile and low-cost pathway to fabricate photocatalysts for viable degradation of organic dye molecules.

## Introduction

The unprecedented demands on available energy supplies, in view of the increasingly severe issues related to the depletion of natural energy resources, drastic climate change, and environmental pollution, dictate to seek for sustainable resources with green energy storage and applications (Arico et al., 2005; Poizot and Dolhem, 2011). Photocatalysis is by far one of the most promising techniques to achieve clean and low-cost light energy conversion and storage (Wang et al., 2009; Gu et al., 2011; Xiang et al., 2015). The development of highly efficient and stable photocatalysts for ultimate use of solar energy in wastewater treatment has been recognized as one of the currently most challenging topics in environmental remediation. For this purpose, cadmium sulfide (CdS), as a typical and important semiconductor, has recently attracted extensive attention due to its great potentials as viable photocatalysts. The suitable band gap energy (2.4 eV) of CdS allows the effective activation under sunlight irradiation (Ma et al., 2007; Zhang et al., 2011) that is superior for applications in many fields including optoelectronics (Li et al., 2010), photovoltaics (Pan et al., 2012), chemical sensors (Ferancová et al., 2010), and photocatalysts (Hu et al., 2010; Liu et al., 2011; Yang et al., 2012). However, the practical usage of CdS in aqueous photocatalysis is seriously impeded by photocorrosion that produces toxic Cd^2+^ ions and increases environmental contamination.

A number of strategies have been put forward to enhance the photocatalytic activity of CdS nanomaterials, such as structural and morphological modification, ion doping, surface sensitization, introduction of cocatalysts, hybridization into coupled semiconductors etc. Deposition of CdS on supports seems to be a facile and effective pathway to fabricate stable composite CdS nanomaterials. In this regard, carbon-based materials i.e., graphene, carbon nanotube, carbon nanofiber, templated porous carbon and activated carbon (Mayrhofer and Arenz, 2009; Park et al., 2012; Wang et al., 2014; Larcher and Tarascon, 2015), have been widely used as carriers in the fabrication of CdS loaded nanomaterials. Besides, the supporting materials offer an ideal platform for the dispersion of photocatalysts that favors effective access of substrates with catalytic active sites. Bera et al. designed a 2D hybrid nanostructure of reduced graphene oxide supported CdS nanosheet, showing superior photocatalytic efficiency toward dye degradation (Bera et al., 2015). Hu and co-workers discovered a carbon-coated CdS nanostructure material, which was found to possess enhanced photostability and photocatalytic activity (Hu et al., 2013). Cao et al. prepared a single-layer graphene-CdS quantum dot nanocomposite which showed promising optoelectronic properties (Cao et al., 2010).

Biomass-derived activated carbon, which is typically obtained from the carbonization of biomass at elevated temperatures followed by activation via physical and/or chemical methods (Guo and Lua, 1999; Yalcin and Sevinc, 2000; Carvalho et al., 2004), represents a class of new porous materials with high specific surface area and porosity. Agricultural and garden wastes have been most widely investigated as biomass resources to prepare biomass-derived activated carbon materials (Subramanian et al., 2007; Kalpana et al., 2009; Olivares-Marin et al., 2009; Huang et al., 2011; Li et al., 2011). Herein, we seek to develop CdS@SAC composite materials by loading CdS nanomaterials on lotus-seedpod-derived activated carbon (SAC). The as-prepared CdS@SAC composite materials have shown enhanced photocatalytic activity toward the degradation of rhodamine B (RhB) in aqueous solutions. It is demonstrated that the porous carbon supports enhance the generation of catalytically active species, as well as the separation of photogenerated electrons and holes. This study provides a green and low-cost method to achieve photocatalysts with high efficiency under visible irradiation.

## Experimental

### Materials

Cadmium acetate dihydrous [Cd(CH_3_COO)_2_·2H_2_O], thioacetamide, ethanediamine, potassium hydrate (KOH), and RhB were purchased commercially as analytical reagents. Reaction solutions and stock solutions were prepared using deionized water supplied with a UPT-I-5T ultrapure water system. Pristine lotus seedpods were collected from a local cultivate field near Nanchang, China.

### Characterization

Scanning electron microscopy (SEM) images were obtained by using a JSM6700-F working at 10 kV. Transmission electron microscopy (TEM) and high resolution TEM (HRTEM) images were recorded by using an FEIT 20 working at 200 kV. Powder X-ray diffraction (PXRD) were carried out on a Miniflex 600 diffractometer with Cu Kα radiation (λ = 0.154 nm). N_2_ adsorption/desorption isotherms and Brunauer-Emmett-Teller (BET) surface area measurements were obtained on a *Micromeritics* ASAP 2460 instrument. The diffuse reflectance spectra (DRS) were recorded in the range of 200 nm to 800 nm on a *Shimadzu* UV-2600 UV-vis spectrophotometer with BaSO_4_ as the background. X-ray photoelectron spectroscopy (XPS) measurements were performed on a *Thermo Fisher* ESCALAB 250Xi spectrometer with Al Kα X-ray source (15 kV, 10 mA). The Electron spin resonance (ESR) spectra were recorded on a *Bruker* A300 spectrometer to detect the generation of active species under visible light irradiation (420 nm < λ < 780 nm).

### Preparation of biomass-derived activated carbon

The naturally dried pristine lotus seedpods were washed carefully with deionized water for three times, dried in oven at 70°C for 24 h, and crushed through a 100 mesh sieve. The biomass was then carbonized in a tubular furnace at 600°C for 4 h with a heating rate of 5°C min^−1^ under N_2_ atmosphere. The resultant carbon material was activated by mixing with KOH in 1:3 ratio (w/w), followed by heating at 600°, 700°, or 800°C for 1 h under N_2_ atmosphere respectively. The collected samples were then washed with 0.1 M HCl and deionized water sequentially until pH of the filtrate was around 7.0 and finally dried at 70°C for 5 h to get the final products. The lotus-seedpod-derived activated carbon materials were denoted as SAC-T, where T was the temperature of activation (Wang et al., 2006; Lang et al., 2014; Li et al., 2017).

### Preparation of CdS nanomaterials

The CdS nanomaterials were prepared referring to a reported method by applying Cd(CH_3_COO)_2_·2H_2_O and thioacetamide as cadmium and sulfur sources (Lang et al., 2014), respectively, and using ethanediamine as the solvent. The as-prepared CdS nanomaterial was collected by filtration, washed with deionized water and ethanol, respectively, for three times, and separated by centrifugation before drying at 70°C for 5 h in oven.

### Preparation of CdS@SAC-T composite materials

CdS nanomaterial (100 mg) and SAC (200 mg) were mixed in 30 mL deionized water under magnetic stirring for 1 h, followed by drying at 105°C for 12 h in oven. The mixture was calcinated in a tubular furnace at 300°C for 1 h under N_2_ atmosphere. The as-prepared CdS@SAC-T composite materials were used later as photocatalysts under visible light irradiation.

### Photocatalytic reaction

Photocatalytic reactions were carried out using a 300 W Xe-arc lamp (PLS-SXE300C, Beijing) as light source which was equipped with 420 and 780 nm cutoff filters to ensure visible light illumination. The degradation of RhB was selected as a model reaction to evaluate the photocatalytic activity of CdS@SAC-T composite materials. In a typical procedure, a portion of composite material photocatalyst (40 mg) was dispersed in a 200 mL Pyrex glass vessel with 100 mL RhB solution (40 mg L^−1^). The suspension was stirred magnetically in dark for 30 min before visible light irradiation. The photocatalytic experiment was then monitored according to the absorbance at 554 nm using a UV-vis spectrophotometer after certain time of intervals upon visible light irradiation. The photocatalytic activity was calculated by the following formula: *R* = *C*/*C*_0_ × 100% where *R* was the degradation rate, *C*_0_ and *C* are the characteristic absorbency of RhB before and after irradiation, respectively.

### Detection of active species

Active species contributing to the photocatalytic activity were identified by scavenger tests and ESR measurements. In order to verify the formation of reactive radicals responsible for RhB oxidation, the 5,′5-dimethyl-1-pyrroline-N-oxide (DMPO) was used as the spin trapper to capture ${\text{O}}_{2}{•}^{-}$. In a typical procedure, 10 mg of composite materials was dispersed in 1.0 mL methanol (for ${\text{O}}_{2}{•}^{-}$) followed by the addition of 20.0 μL DMPO. The above mixture was then irradiated with 300 W Xe lamp for 0–60 min and analyzed by using ESR technique. Hydroxyl radicals (OH•) produced during the photocatalysis under visible light was estimated by the fluorescence method using terephthalic acid (TA) as a probe molecule. In a typical process, 10 mg of photocatalyst was dispersed in 100 mL of 5.0 × 10^−4^ M TA and diluted aqueous NaOH (2.0 × 10^−3^ M) solution. The resulting suspension was then exposed to visible light irradiation. At regular intervals, 2.5 mL of the suspension was collected and filtrated (0.22 micron filter) to measure the maximum fluorescence emission intensity with an excitation wavelength of 315 nm. The fluorescence signals of 2-hydroxyterephthalic acid (TAOH) at 425 nm were recorded (Bera et al., 2015).

## Results and discussion

**Scheme 1:**
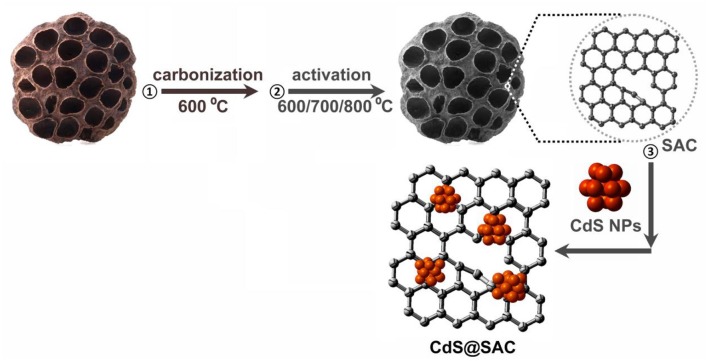
Synthetic pathway of the CdS@SAC composite materials.

Pristine lotus seedpods were firstly carbonized at 600°C in N_2_ atmosphere to afford biomass-derived carbon materials. After that, the carbon were activated at various temperatures to produce SACs (SAC-T; T = 600, 700, and 800) in the presence of KOH. The CdS@SAC-T composite materials were synthesized by loading CdS nanomaterials onto SACs via calcinations of the mixture at 300°C. Detailed synthetic pathway is illustrated in Scheme 1. The as-prepared CdS@SAC-T composite materials were characterized by a variety of physical characterization techniques including powder X-ray diffraction (PXRD), XPS, SEM, and transmission electron microscopy (TEM) etc. to fully identify their microstructure, as well as the surface morphology and functionality.

**Figure 1:**
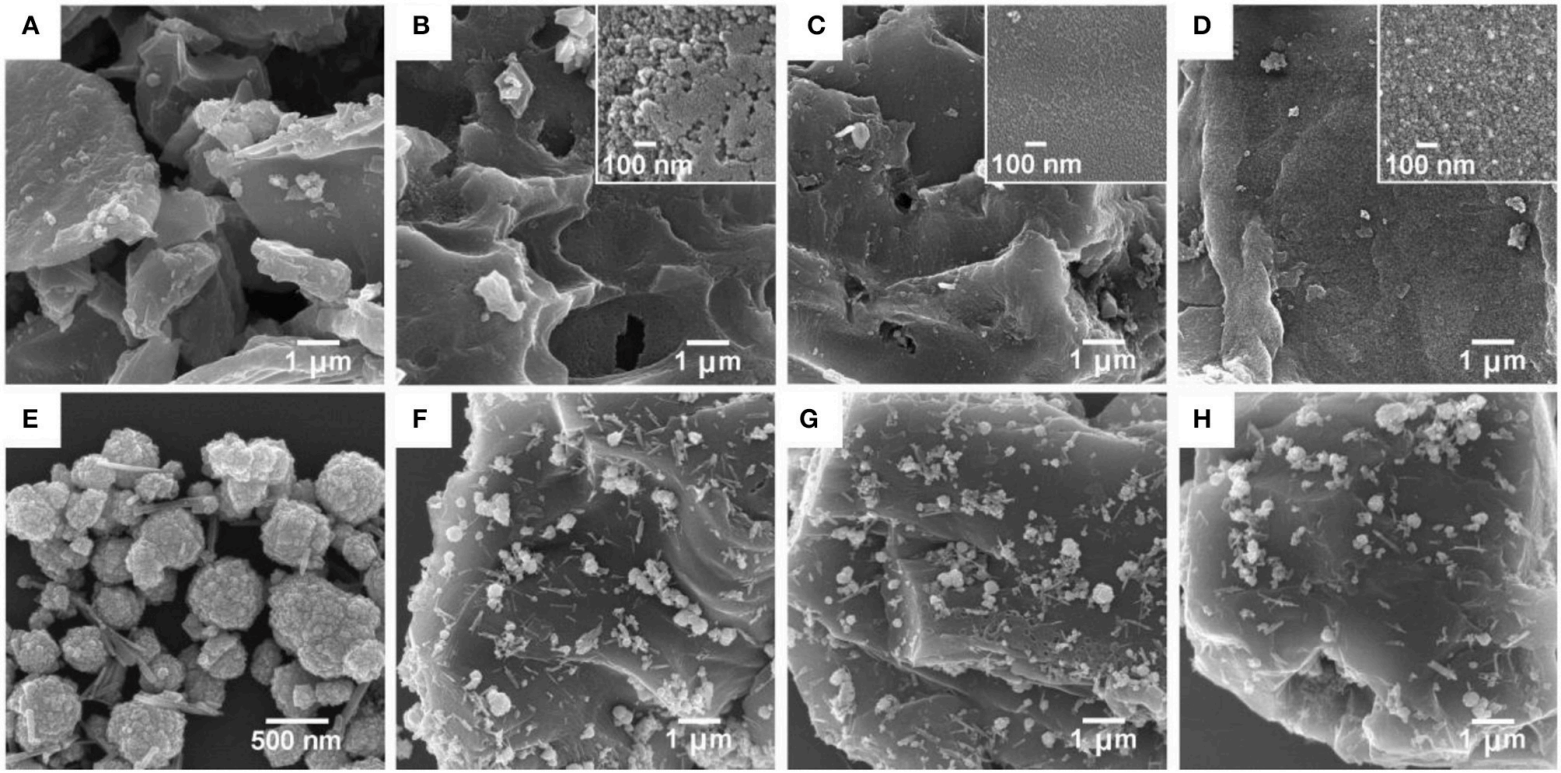
SEM images. **(A)** Pristine SC; **(B)** SAC-600 (inset: magnified image); **(C)** SAC-700 (inset: magnified image); **(D)** SAC-800 (inset: magnified image); **(E)** CdS precursor; **(F)** CdS@SAC-600; **(G)** CdS@SAC-700; and **(H)** CdS@SAC-800.

Scanning electron microscopy (SEM) images of the pristine lotus-seedpod-derived carbon material (SC), activated carbon (SAC-T), and composite materials (CdS@SAC-T) are shown in Figure 1. It is clear that the SC material derived from the pristine lotus seedpods exhibits considerably rough and hierarchical surface (Figure 1A). Moreover, the SAC materials possess surfaces with nanosized particles after chemical activations (Figures 1B–D), and higher temperature of activation seems to produce smoother surface morphology of the carbonaceous material (SAC-800, Figure 1D). The CdS precursor, prepared via solvothermal reactions, displays a major phase of nanospheres and a minor phase of nanorods (Figure 1E), which is in consistent with the literature (Lang et al., 2014). Composite materials are generally formed upon loading of CdS nanomaterials on these SAC supports (Figures 1F–H). Chemical composition of the CdS@SAC-T composite materials was later detected by Inductive Coupled Plasma (ICP) measurements, and a moderate and consistent CdS content ranging from ca. 29.4 to ca. 32.5% (Table 1) was obtained.

**Table 1:** Elemental analyses, BET surface areas, and mean pore diameters of various carbonaceous and composite materials.

**Samples**	**Contents**				**S_BET_ (m^2^ g^−1^)**	**D (nm)**
	**C (%)**	**H (%)**	**S (%)**	**Cd (%)**		
SC	74.3	2.4	n.a.	n.a.	2.1	n.a.
SAC-600	68.1	4.9	n.a.	n.a.	956.9	2.2
SAC-700	68.6	4.4	n.a.	n.a.	1154	2.3
SAC-800	68.3	4.6	n.a.	n.a.	1539	2.5
CdS@SAC-600	35.9	2.0	7.5	24.8	459.7	1.7
CdS@SAC-700	35.4	1.9	6.3	23.1	766.6	1.6
CdS@SAC-800	38.2	0.57	6.9	25.6	943.2	1.9

**Figure 2:**
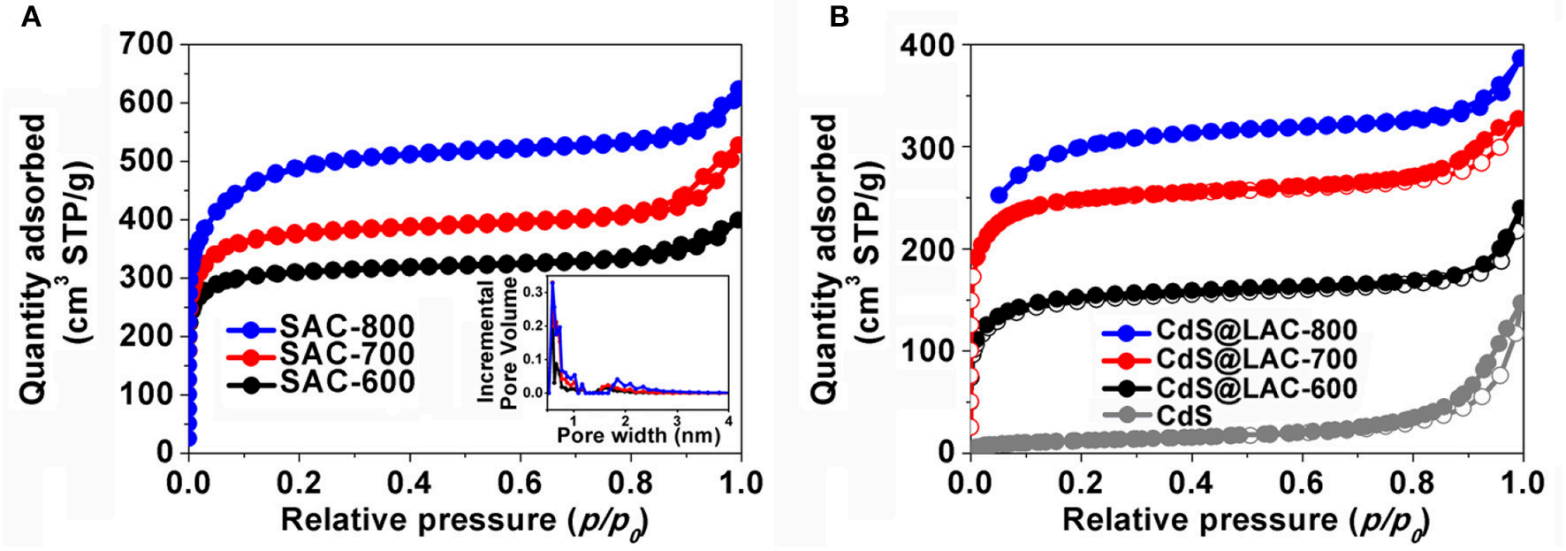
N_2_ adsorption/desorption isotherms of **(A)** SAC-T, inset: pore size distribution; and **(B)** CdS precursor and CdS@SAC-T composite materials.

In order to identify the microporosity of SAC-T and CdS@SAC-T materials, N_2_ adsorption isotherms at 77 K were recorded. The pristine SC shows negligible N_2_ adsorption which suggests the nonporous characteristics of the material (Table 1). Adsorption/desorption isotherms of SAC-T revealed the reversible N_2_ uptake and suggested the typical type-I adsorption/desorption behaviors (Figure 2A). The Brunauer-Emmett-Teller (BET) surface areas of SAC-T are 956.9, 1,154, 1,539 m^2^·g^−1^ (Table 1), which are considerably high in biomass-derived activated carbon materials (Figure 2A). Pore size distribution calculations suggest the average pore diameters of SAC-T are 2.2, 2.3, and 2.5 nm with dominant microporosity below 2.0 nm (Figure 2A, inset). These results clearly indicate that, the activation with KOH is effective in creating microstructures and porosity for SAC-T. In addition, the surface areas of SAC-T materials are further enhanced with the increase of activation temperatures (Table 1, Figure 2A). Furthermore, rational decrease in BET surface areas and average pore sizes for CdS@SAC-T composite materials are observed after CdS loading, as shown in Figure 2B, which indicates the successful deposition of CdS nanomaterials on SAC-T supports.

**Figure 3:**
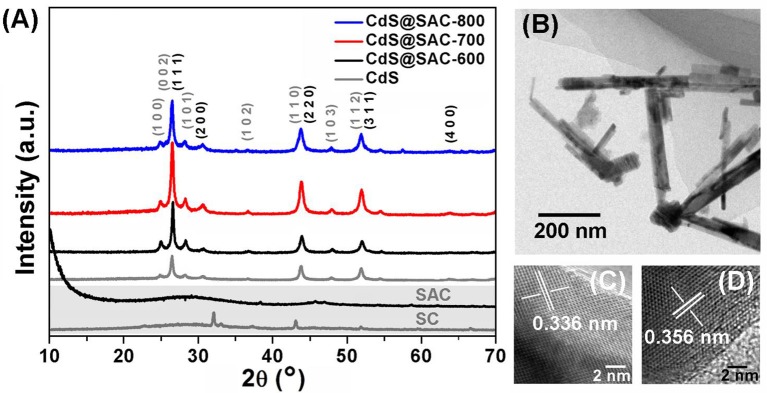
**(A)** PXRD patterns of the SC, SAC, CdS nanomaterial, and CdS@SAC-T composite materials; and typical TEM **(B)** HRTEM images of CdS@SAC-800 **(C,D)**.

PXRD was used to identify the phase characteristics and XPS was employed to discover the surface functionality of the CdS@SAC-T materials. In the PXRD of pristine SC and SAC carbonaceous materials, the dispersion peaks centered at 23° and 43° are ascribed to the graphitized carbon. The sharp peaks observed in SC can be ascribed to the characteristics of crystalline minerals i.e., CaCO_3_ and CaMg(CO_3_)_2_ (Cao and Harris, 2010; Wu et al., 2012; Cho et al., 2017). The PXRD patterns of CdS@SAC-T are similar to that of the CdS nanomaterials with crystalline diffraction peaks that are fully indexed to the mixed phases of cubic (JCPDS 10-0454) and hexagonal (JCPDS 41-1049) CdS (Figure 3A; Dai et al., 2014; Li et al., 2016). Moreover, crystalline of the CdS@SAC-T is generally better than that of the CdS precursor. TEM (Figure 3B) and HRTEM images identify that the supported CdS nanomaterials are in crystalline phases with crystal plane spacing of 0.336 and 0.356 nm (Figures 3C,D), which correspond to the (1 1 1) lattice plane of cubic phase and the (1 0 0) lattice plane of hexagonal phase of CdS, respectively.

**Figure 4:**
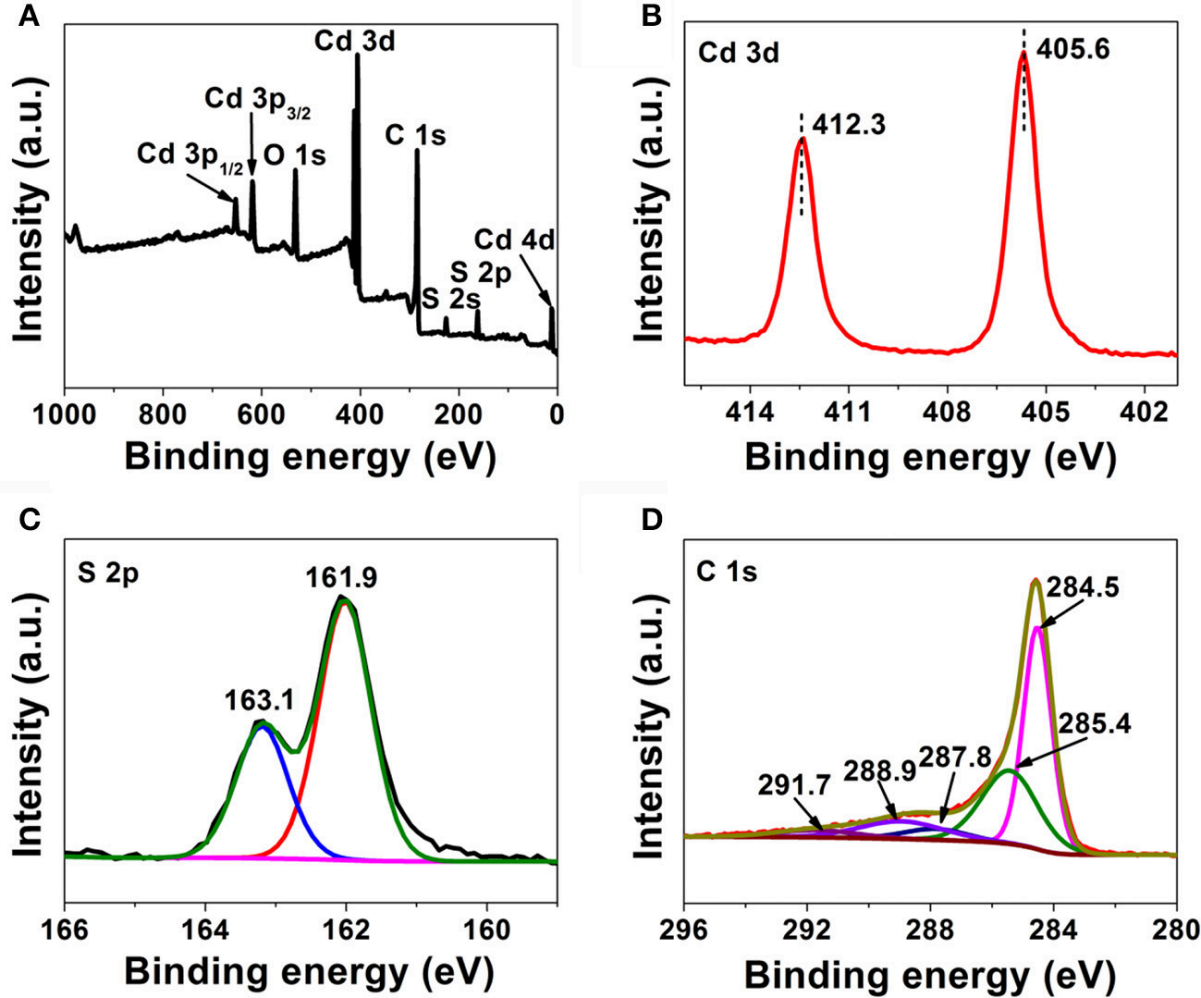
XPS survey spectrum of the CdS@SAC-800 composite material **(A)** and high-resolution spectra of Cd 3d **(B)**, S 2p **(C)**, and C 1s **(D)**.

In a typical XPS spectra (Figure 4A, CdS@SAC-800), the doublet characteristics at 405.6 eV (Cd 3d_5/2_) and 412.3 eV (Cd 3d_3/2_) are assigned to the Cd^2+^ species (Figure 4B). The two peaks at 161.9 (S 2p_3/2_) and 163.1 eV (S 2p_1/2_) in the S 2p spectrum (Figure 4C) are from the spin-orbit doublet of S^2−^, respectively. Moreover, the atomic ratio of Cd^2+^ and S^2−^ is estimated to be approximately 1:1, which is a confirmation of the successful loading of CdS on the SAC supports. The C 1s peaks at 284.5, 285.4, 287.8, 288.9, and 291.7 eV are assigned to C = C, C–O, C = O, COOR and π-π^*^ (Figure 4D), respectively, demonstrating the presence of multiple functionality in SAC supports (Chen et al., 2007; Li et al., 2007; Yu et al., 2014).

**Figure 5:**
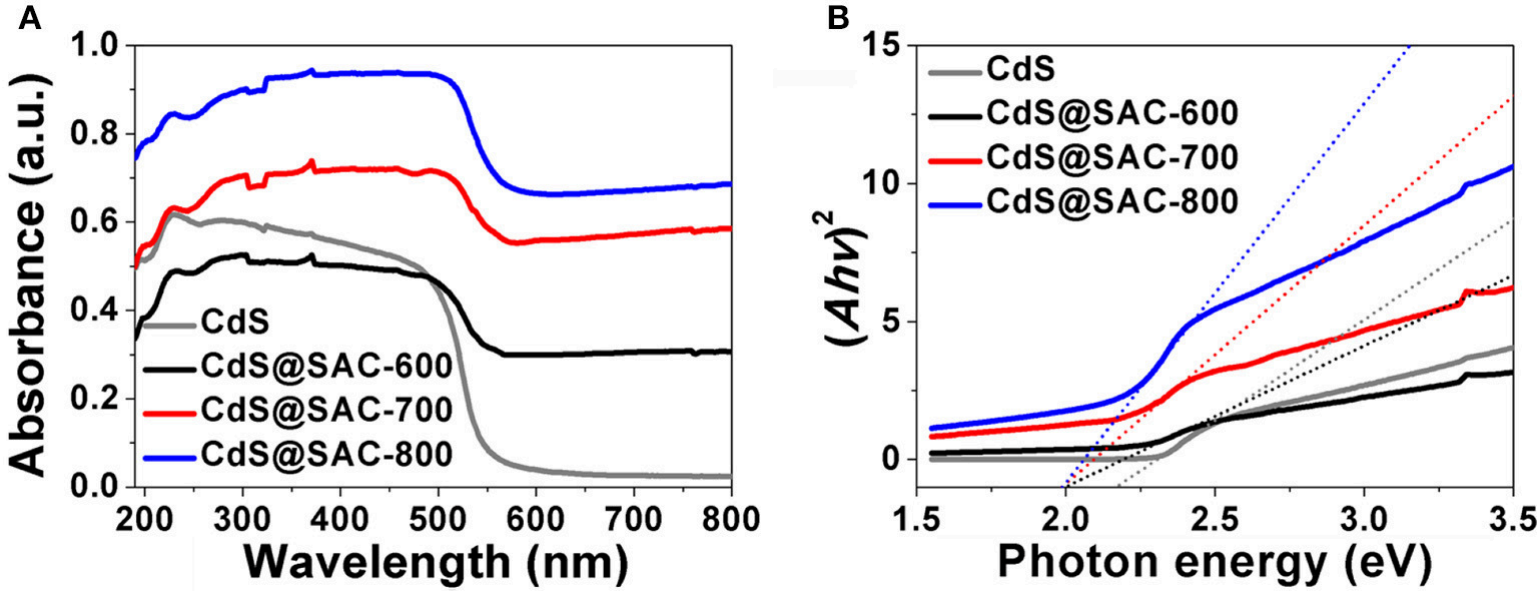
UV-vis DRS spectra **(A)** and K-M plots **(B)** of the CdS precursor and CdS@SAC-T composite materials.

Diffuse reflectance spectra (DRS) of CdS@SAC-T composite materials displayed broad absorption bands in the visible light and near infrared regions with clear red shifts in comparison with that of the CdS nanomaterials (Figure 5A). Band gap energy of the CdS@SAC-T composite materials are determined to be approximately 2.02, 2.01, and 1.99 eV (Figure 5B), respectively, which are significantly lower than that of the CdS precursor (2.22 eV). The similar band gap energy observed for CdS@SAC-T suggests a comparable light-harvesting efficiency of these composite materials. Therefore, the absorbance of visible light is expected to improve the photocatalytic activity of CdS@SAC-T composite materials.

**Figure 6:**
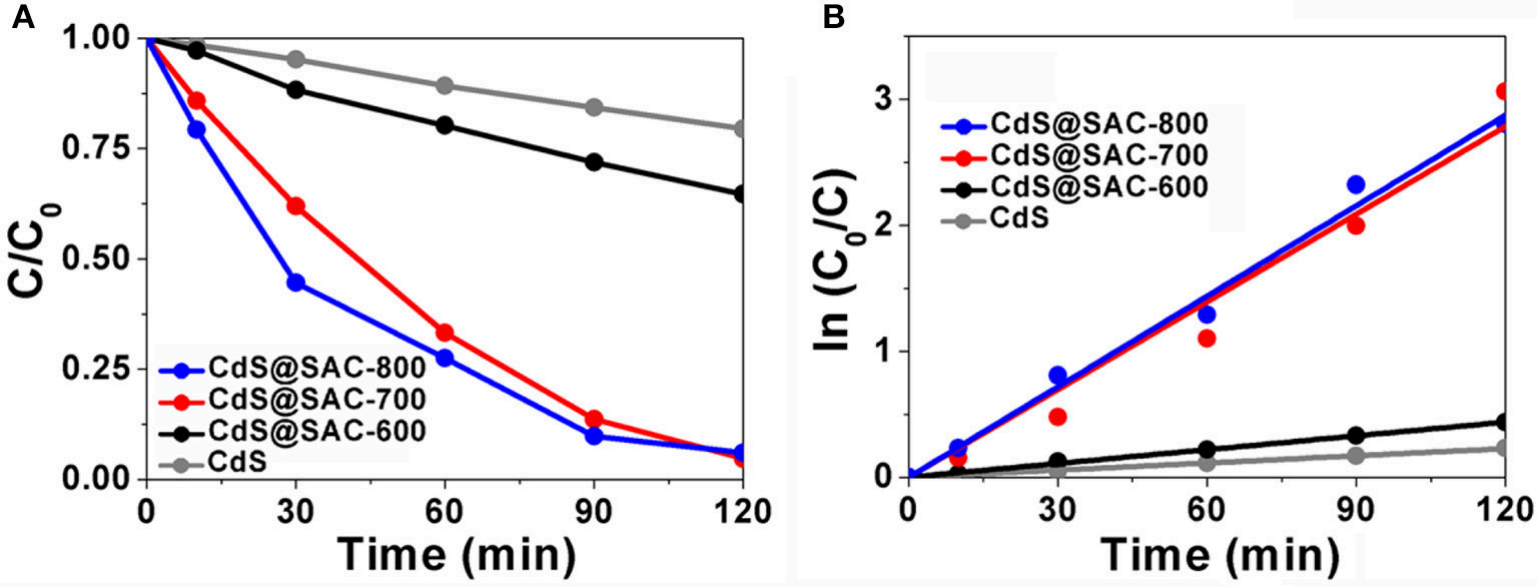
**(A)** Photocatalytic degradation of RhB, and **(B)** plot of ln (*C*_0_/*C*) as a function of irradiation time in the presence of various photocatalysts.

Photocatalytic activity of CdS@SAC-T composite materials was evaluated by the degradation of RhB under visible light irradiation as a model reaction. The photocatalytic efficiency of CdS@SAC-T material photocatalysts is generally higher than that of CdS precursor (Figure 6A), following an order of CdS@SAC-800 > CdS@SAC-700 > CdS@SAC-600. Moreover, the enhanced photocatalytic activity of the composite materials might be attributable to the porous nature of CdS@SAC-T photocatalysts in which the catalytically active CdS nanoparticles are well dispersed. Meanwhile, the high specific surface area of photocatalysts favors the enrichment of RhB substrates, and so to improve the photocatalytic efficiency. In order to quantitatively understand the reaction kinetics of the RhB degradation (Figure 6B), we applied the pseudo-first order model to obtain rate constant (κ) (Li et al., 2006; Jing et al., 2017). It is clear that CdS@SAC-800 exhibits the best photocatalytic degradation efficiency (ca. 2.4 × 10^−2^ min^−1^), which is approximately 13 times higher than that of the CdS nanomaterial (ca. 1.9 × 10^−3^ min^−1^). More specifically, the photocatalytic efficiency of CdS@SAC-800 reaches ca. 94.0% within 120 min. In comparison, the CdS nanomaterial itself exhibits considerably lower degradation efficiency of ca. 20.5% under the same conditions.

**Figure 7:**
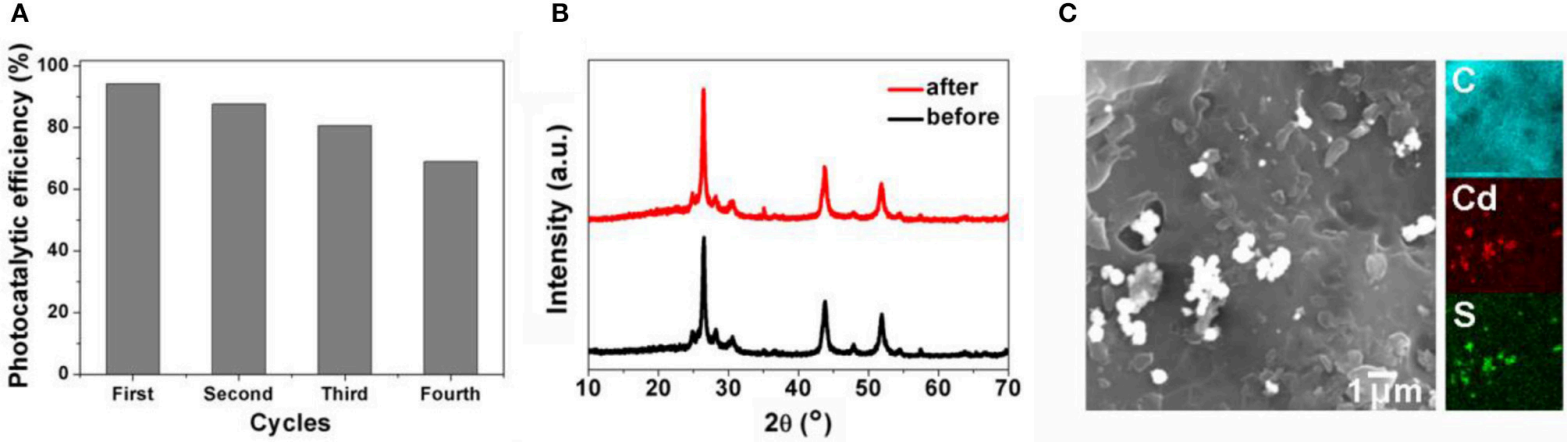
**(A)** Photocatalytic efficiency of CdS@SAC-800 in recycling reaction of RhB degradation; **(B)** PXRD patterns and **(C)** SEM of CdS@SAC-800 before and after recycling reactions.

The reusability of CdS@SAC-800 was demonstrated by recycling reactions of RhB degradation under visible light. It has been found that the CdS@SAC-800 is able to maintain more than 70% of the initial photocatalytic efficiency ability after four cycles (Figure 7A). The reasonable decrease of photocatalytic efficiency might be originated from the insignificant loss of photocatalysts during the recovery processes. In addition, PXRD patterns of the CdS@SAC-800 photocatalyst before and after recycling reactions were nearly identical (Figure 7B), suggesting the structural stability of the photocatalyst, which is promising for its practical applications. SEM images shown in Figure 7C indicated that morphology of CdS@SAC-800 was nearly unchanged during the recycling reactions.

**Figure 8:**
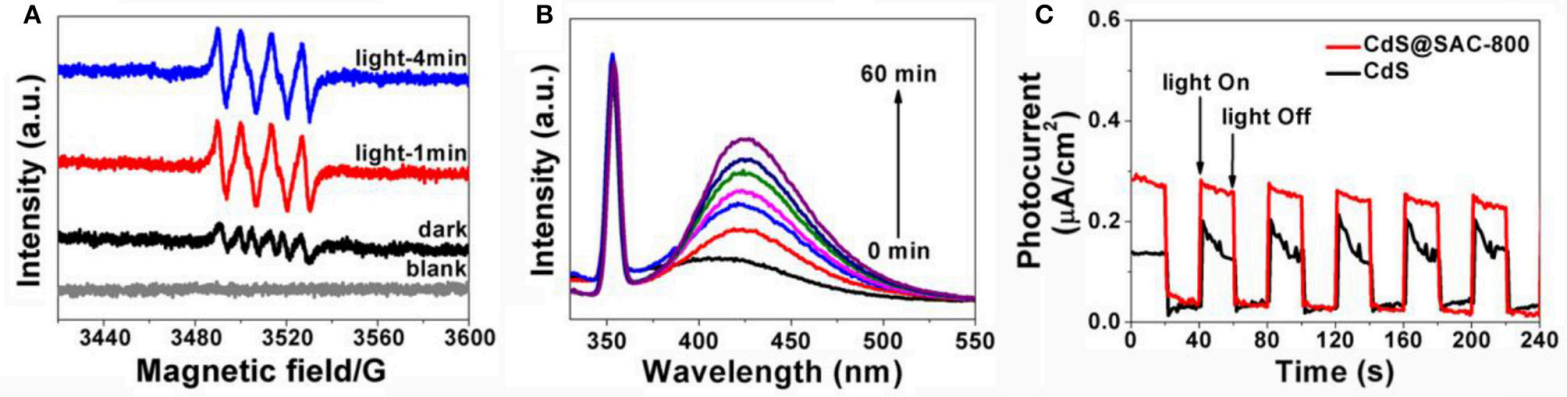
**(A)** DMPO spin-trapping ESR spectra for the CdS@SAC-800 composite material under visible light (420 nm < λ < 780 nm) irradiation in methanol dispersion for DMPO-${\text{O}}_{2}{•}^{-}$; **(B)** the time-dependent fluorescence intensity of TAOH the presence of CdS@SAC-800 under visible irradiation; **(C)** transient photocurrent response of CdS and CdS@SAC-800 under visible irradiation.

Further study by using ESR spin-trapping technique with DMPO was performed to detect the ${\text{O}}_{2}{•}^{-}$ radicals (Meng et al., 2011; Lin et al., 2012). Upon visible light irradiation, CdS@SAC-800 material generates stronger characteristic peaks for DMPO-${\text{O}}_{2}{•}^{-}$ species, which also supports the hypothesis of dominant ${\text{O}}_{2}{•}^{-}$ species present in this photocatalytic system (Figure 8A). The formation of OH• during the photocatalysis was detected by the fluorescence technique using terephthalic acid (TA) as a probe regent, which readily reacts with OH• to produce the highly fluorescent product 2-hydroxyterephthalic acid (TAOH). The fluorescence intensity of TAOH is proportional to the amount of OH• produced on the surface of photocatalysts. The maximum emission intensity in fluorescence spectra was recorded at 425 nm by the excitation at 315 nm. Moreover, the fluorescence intensity increases gradually with irradiation time (Figure 8B), showing a positive correlation with the formation rate of OH• radicals. Photocatalysis in this current system is highly energy-efficient due to the introduction of porous carbonaceous supports that increase interface contact between the CdS and carbon support. Therefore, the efficient contact of photocatalysts and organic substrates is crucial. Moreover, photocatalytic ability of the composite materials depends largely on the microporous carbonaceous supports which favor the effective generation and transportation of photongenerated active species such as *h*^+^, OH•, and ${\text{O}}_{2}{•}^{-}$. The generation of photocurrent in the photocatalyst is illustrated in Figure 8C. Upon visible irradiation, a photocurrent is tested within the time intervals of 20 s, which decreases rapidly to the initial levels as long as light is off. The photocurrent of CdS@SAC-800 is enhanced and more stable than that of CdS precursor. It is clear that the electron-hole separation is more efficient in the composite system.

**Scheme 2:**
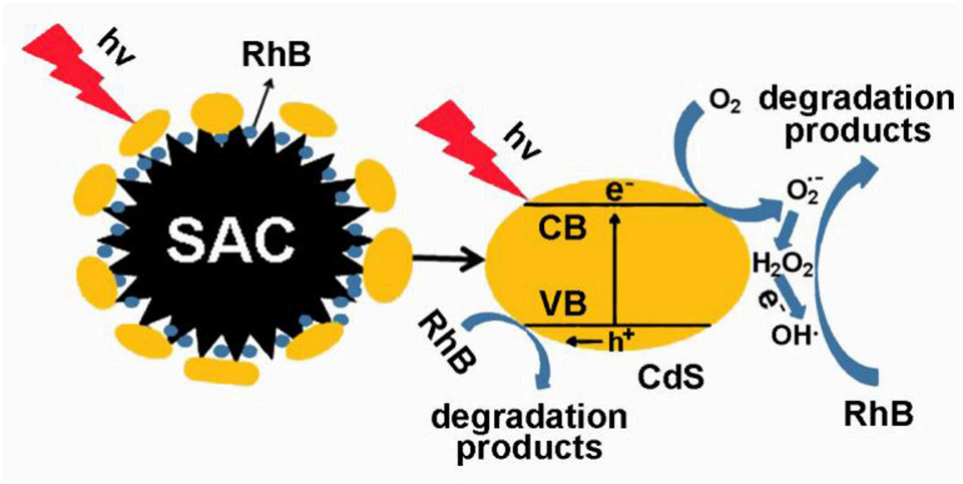
Proposed photodegradation mechanism for CdS@SAC-T composite materials as photocatalysts under visible irradiation.

Based on the above observations, a probable visible-light-driven photocatalysis mechanism is proposed (Scheme 2). During photocatalysis, electrons transfer from the valence bands to the conduction bands of CdS upon irradiation of visible light (Zhu et al., 2016, 2017). Thus, the photogenerated carriers take part in the generation of active species such as *h*^+^, OH•, and ${\text{O}}_{2}{•}^{-}$ etc. On the other side, oxidative holes in the valence bands attack RhB by capturing electrons and degradation occurs. Overall, dye molecules are degraded by the active species as follows:

$$\begin{array}{l} \text{CdS}+\text{hv}\to \text{CdS}({\text{e}}_{\text{CB}}^{-}\ldots {\text{h}}_{\text{VB}}^{+})\\ \text{CdS}({\text{e}}_{\text{CB}}^{-})\text{+SAC}\to \text{SAC}(\text{e})\text{+CdS}\\ \text{SAC}(\text{e}){+O}_{2}\to {\text{O}}_{2}{•}^{-}\text{+SAC}\\ {\text{O}}_{2}{•}^{-}{\text{+H}}_{2}\text{O}\to {\text{HO}}_{2}•{\text{+OH}}^{-}\\ {\text{HO}}_{2}•{\text{+H}}_{2}\text{O}\to \text{OH}•{\text{+H}}_{2}{\text{O}}_{2}\\ {\text{H}}_{2}{\text{O}}_{2}\to \text{2OH}•\\ \text{CdS}({\text{h}}_{\text{VB}}^{\text{+}}),{\text{O}}_{2}{•}^{-},\text{OH}•\text{etc}.\text{+RhB}\to \text{photodegradation of RhB} \end{array}$$

## Conclusion

In summary, CdS@SAC-T composite materials based on cadmium sulfite nanoparticles and SAC supports have been successfully prepared and applied to the photodegradation of RhB. The composite materials have exhibited enhanced photocatalytic efficiency compared with the CdS precursor. Of special notice, the introduction of porous carbonaceous supports has been demonstrated to be responsible for the highly energy-efficient photocatalysis, by which the interface contact between CdS and carbon supports is increased and so to provide efficient contact of the photocatalysts and organic substrates. Moreover, photocatalytic ability of the composite materials depends largely on the microporous carbonaceous supports which favor the effective generation and transportation of photongenerated active species such as *h*^+^, OH•, and ${\text{O}}_{2}{•}^{-}$. This research protocol provides a facile means for the recycle and reuse of agricultural wastes as well as a low-cost pathway to fabricate photocatalysts for viable degradation of organic pollutants.

## Author contributions

JL, H-LC, and H-BH designed the research. H-BH, YW, F-YC, and W-BJ conducted experiments and characterized the materials. NZ and CL conducted the catalysis. JL, H-LC, H-BH, and F-YC co-wrote the paper.

### Conflict of interest statement

The authors declare that the research was conducted in the absence of any commercial or financial relationships that could be construed as a potential conflict of interest.
